# Novel ^68^Ga-Labeled Pyridine-Based Fibroblast Activation Protein-Targeted Tracers with High Tumor-to-Background Contrast

**DOI:** 10.3390/ph16030449

**Published:** 2023-03-16

**Authors:** Arsyangela Verena, Hsiou-Ting Kuo, Helen Merkens, Jutta Zeisler, Shreya Bendre, Antonio A. W. L. Wong, François Bénard, Kuo-Shyan Lin

**Affiliations:** 1 Department of Molecular Oncology, BC Cancer Research Institute, Vancouver, BC V5Z 1L3, Canada; 2 Department of Radiology, University of British Columbia, Vancouver, BC V5Z 1M9, Canada; 3 Department of Functional Imaging, BC Cancer, Vancouver, BC V5Z 4E6, Canada

**Keywords:** fibroblast activation protein, FAP inhibitors, PET/CT, Gallium-68, pyridine-based pharmacophore, FAPI-04

## Abstract

Compared to quinoline-based fibroblast activation protein (FAP)-targeted radiotracers, pyridine-based FAP-targeted tracers are expected to have faster pharmacokinetics due to their smaller molecular size and higher hydrophilicity, which we hypothesize would improve the tumor-to-background image contrast. We aim to develop ^68^Ga-labeled pyridine-based FAP-targeted tracers for cancer imaging with positron emission tomography (PET), and compare their imaging potential with the clinically validated [^68^Ga]Ga-FAPI-04. Two DOTA-conjugated pyridine-based AV02053 and AV02070 were synthesized through multi-step organic synthesis. IC_50_(FAP) values of Ga-AV02053 and Ga-AV02070 were determined by an enzymatic assay to be 187 ± 52.0 and 17.1 ± 4.60 nM, respectively. PET imaging and biodistribution studies were conducted in HEK293T:hFAP tumor-bearing mice at 1 h post-injection. The HEK293T:hFAP tumor xenografts were clearly visualized with good contrast on PET images by [^68^Ga]Ga-AV02053 and [^68^Ga]Ga-AV02070, and both tracers were excreted mainly through the renal pathway. The tumor uptake values of [^68^Ga]Ga-AV02070 (7.93 ± 1.88%ID/g) and [^68^Ga]Ga-AV02053 (5.6 ± 1.12%ID/g) were lower than that of previously reported [^68^Ga]Ga-FAPI-04 (12.5 ± 2.00%ID/g). However, both [^68^Ga]Ga-AV02070 and [^68^Ga]Ga-AV02053 showed higher tumor-to-background (blood, muscle, and bone) uptake ratios than [^68^Ga]Ga-FAPI-04. Our data suggests that pyridine-based pharmacophores are promising for the design of FAP-targeted tracers. Future optimization on the selection of a linker will be explored to increase tumor uptake while maintaining or even further improving the high tumor-to-background contrast.

## 1. Introduction

Fibroblast activation protein (FAP) is a type II transmembrane serine protease and has both dipeptidyl peptidase and endopeptidase activity [1,2]. The serine residue in FAP acts as a nucleophile, cleaving Pro-X peptide bonds, where X is any amino acid except proline or hydroxyproline [3]. FAP is found to be highly expressed in activated fibroblasts, such as during wound healing [4], and is essentially absent in normal adult cells [5]. Studies have shown that FAP is highly expressed in 90% of epithelial tumors, including prostate, pancreatic, colon, and breast cancers [6,7,8], and is expressed by the cancer-associated fibroblasts (CAFs) in the tumor microenvironment (TME) [9,10]. Overexpression of FAP in tumors has been shown to promote tumor growth, angiogenesis [11], and metastasis [12]. Silencing FAP has been shown to induce tumor cell apoptosis [13], indicating its important role in cancer cells survival. Moreover, a high level of FAP expression in the stroma is associated with aggressive disease progression and recurrence in colorectal cancer [14]. Therefore, FAP is a promising target for cancer diagnosis and therapy.

Many FAP inhibitors have been developed as potential anticancer agents, in the form of small molecules, an antibody, and a vaccine [15,16,17,18]. For small molecule-based inhibitors, there are two major groups, which are 2-cyanopyrrolidine derivatives and 2-pyrrolidinylboronic acid derivatives (boroPro). Jansen and co-workers synthesized and compared several (4-quinolinoyl)glycyl-2-cyanopyrrolidine derivatives as FAP inhibitors [19,20]. They observed that difluoro substitution at the 4-position of 2-cyanopyrrolidine ring improved FAP binding affinity, and substituting glycine with other amino acids decreased binding affinity. Therefore, (4-quinolinoyl)glycyl-(4,4-difluoro-2- cyanopyrrolidine) was identified as an optimized pharmacophore for FAP-targeting. Poplawski and co-workers synthesized and compared several boroPro derivatives for binding to FAP [21]. Their data showed that (pyridine-4-carbonyl)-Gly-boroPro had the highest binding affinity (IC_50_(FAP) = 0.47 nM). Replacing Gly with D-Ala reduced the binding affinity to FAP but improved the selectivity of FAP toward other closely related enzymes, including dipeptidyl peptidases IV (DPPIV), 8 (DPP8), and 9 (DPP9), and prolyl oligopeptidase (PREP). Replacing the pyridine-4-carbonyl moiety with a pyridine-3-carbonyl moiety also led to derivatives with reduced FAP binding affinity.

**Figure 1:**
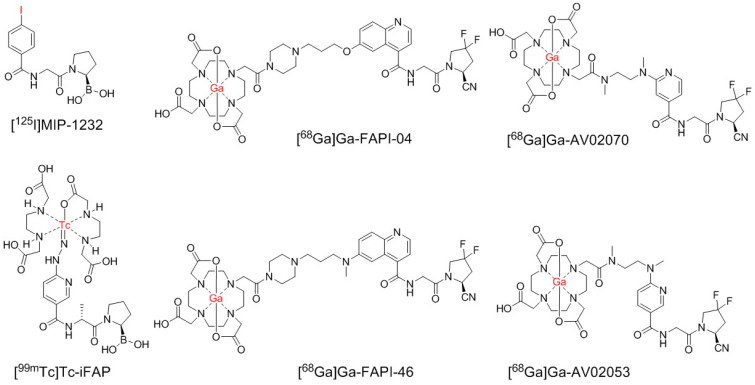
Chemical structures of ^68^Ga-labeled AV02053 and AV02070, and previously reported [^125^I]MIP-1232, [^99m^Tc]Tc-iFAP, and ^68^Ga-labeled FAPI-04 and FAPI-46. The radiolabels are in red.

The first radiolabeled small-molecule FAP inhibitor (FAPI) for imaging was reported in 2015 by Meletta and co-workers, in which a boroPro-based FAPI, [^125^I]MIP-1232 (Figure 1), was synthesized and evaluated for atherosclerotic plaque imaging [22]. The authors concluded that [^125^I]MIP-1232 might not be suitable for plaque imaging as the difference in FAP expression between normal arteries and plaques was modest. However, due its high staining intensity from an autoradiography study on FAP-positive SK-Mel-187 human melanoma tumor xenografts, it might be a promising tool for oncology imaging. Since then, many new radiolabeled FAPIs have been developed and evaluated for cancer imaging and therapy in the clinic. For imaging purposes, [^68^Ga]Ga-FAPI-04 (Figure 1) is the most widely used FAP-targeted tracer and has been shown to have good uptake in several malignancies [23]. In addition, [^68^Ga]Ga-FAPI-04 has been shown to have better biodistribution, tumor-to-background contrast, and faster kinetics compared to [^18^F]FDG [24]. However, [^68^Ga]Ga-FAPI-04 has a relatively short tumor retention, making it unsuitable for theranostic purposes. Therefore, many other promising FAP-targeted radioligands are being developed and some are being evaluated clinically, such as [^177^Lu]Lu-FAP-2286 [25] and [^68^Ga]Ga-FAPI-46 (Figure 1) [26].

Despite promising utilities, there are some pitfalls in FAP-targeted tracers, as non-tumor related uptake of [^68^Ga]Ga-FAPI-04 and [^68^Ga]Ga-FAPI-46 by degenerative lesions associated with joints and vertebral bones, muscles, scars, head-and-neck, and mammary glands has been reported [27]. Therefore, there might be unspecific and off-target uptake of quinoline-based tracers, which can increase the background uptake level and lower the image contrast, thus interfering the identification of FAP-positive tumor lesions and even leading to false diagnoses [28,29]. To improve the tumor-to-background image contrast, we need to develop tracers that can be rapidly excreted from the body, but specifically bind to and be retained in FAP-expressing tumors. Currently, the development of FAP-targeted radioligands focuses on the use of quinoline-based pharmacophores, such as the one in ^68^Ga-FAPI-04 and ^68^Ga-FAPI-46 (Figure 1). Compared with quinoline, pyridine is smaller, more hydrophilic, and could have a faster pharmacokinetics in vivo. Hence, we hypothesized that the pyridine-based FAP-targeted tracers might have faster background clearance and better imaging contrast than the current clinically used quinoline-based FAP-targeted tracers.

Previously, Trujillo-Benítez and co-workers developed a ^99m^Tc-labeled boroPro-based tracer, [^99m^Tc]Tc-HYNIC-D-alanine-boroPro ([^99m^Tc]Tc-iFAP) (Figure 1), which has minimal background (blood, muscle, and bone) uptake and good uptake (5.18 ± 0.82%ID/g) in Hep-G2 tumor xenografts at 2 h post-injection [30]. This demonstrates that a pyridine-based FAP-targeted tracer has the potential to achieve good tumor uptake, a low background, and improved tumor-to-background contrast.

Here, we report the design, synthesis, and evaluation of two pyridine-based FAP-targeted tracers, [^68^Ga]Ga-AV02053 and [^68^Ga]Ga-AV02070 (Figure 1), based on the 4,4-difluoro-2-cyanopyrrolidine pharmacophore. Instead of boroPro in [^99m^Tc]Tc-iFAP, we chose the 4,4-difluoro-2-cyanopyrrolidine pharmacophore as it is easier to synthesize. Instead of D-Ala in [^99m^Tc]Tc-iFAP, we chose Gly as it led to derivatives with a higher binding affinity [21]. The tertiary amine linkage between the linker and pyridine in [^68^Ga]Ga-AV02053 and [^68^Ga]Ga-AV02070 was to mimic the hydrazine group in [^99m^Tc]Tc-iFAP, in which the nitrogens in hydrazine were reported to interact with FAP and improve the binding affinity. The difference between the chemical structures of [^68^Ga]Ga-AV02053 and [^68^Ga]Ga-AV02070 is the position of the carbonyl group to the pyridine nitrogen, which is at the meta position for [^68^Ga]Ga-AV02053 and the para position for [^68^Ga]Ga-AV02070 (Figure 1). The meta position in [^68^Ga]Ga-AV02053 is to mimic the pyridine ring structure of [^99m^Tc]Tc-iFAP. Previously, it was shown that the pharmacophores with the carbonyl group in the para position to the pyridine nitrogen have higher binding affinity and specificity to FAP compared to in the meta position [19]. Therefore, we designed [^68^Ga]Ga-AV02070 to compare with [^68^Ga]Ga-AV02053. The potential of [^68^Ga]Ga-AV02053 and [^68^Ga]Ga-AV02070 for cancer imaging was evaluated by in vitro enzymatic assay, PET imaging, and ex vivo biodistribution studies using a preclinical tumor model, HEK293T:hFAP [31]. The results were then compared with those previously obtained using [^68^Ga]Ga-FAPI-04 [31].

## 2. Results

### 2.1. Synthesis of ^68^Ga/^nat^Ga-Labeled DOTA-Conjugated FAP-Targeted Agents Based on Pyridine-Based Pharmacophores

**Scheme 1:**
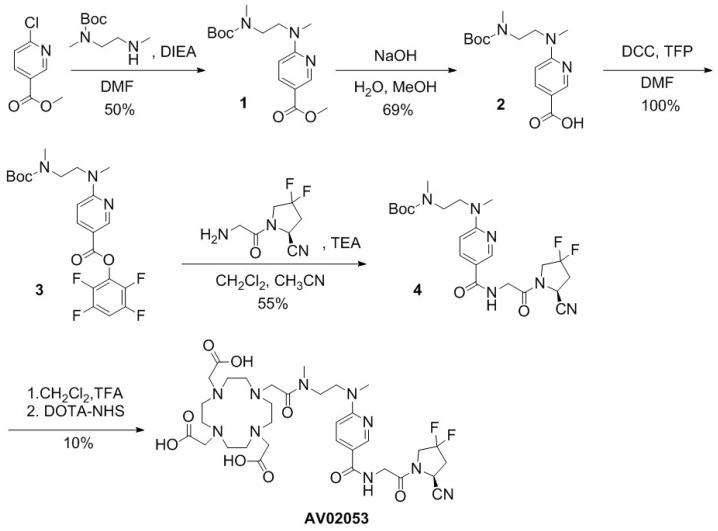
Synthesis of AV02053.

**Scheme 2:**
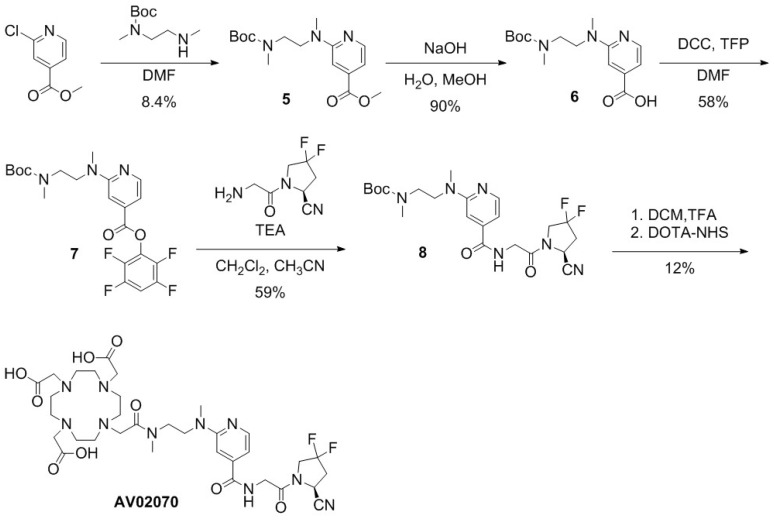
Synthesis of AV02070.

The syntheses of DOTA-conjugated AV02053 and AV02070 are depicted in Scheme 1; Scheme 2, respectively, and detailed synthetic procedures and characterizations for the final products and intermediates are provided in the Supplementary Information. For the preparation of AV02053 (Scheme 1), methyl 6-chloronicotinate was coupled with *N*-Boc-*N*,*N*′-dimethyl-1,2-diaminoethane via nucleophilic substitution to obtain **1** in 50% yield. Compound **2** was obtained in 69% yield by the hydrolysis of the methyl ester in compound **1** with NaOH in a mixture of water and methanol. Esterification of compound **2** with 2,3,5,6-tetrafluorophenol (TFP) led to compound **3** in 100% yield. Compound **4** was obtained in 55% yield by coupling the activated ester **3** with (*S*)-1-(2-aminoacetyl)-4,4-difluoropyrrolidine-2-carbonitrile [20]. *Tert*-butyloxycarbonyl (Boc) protecting group was removed using trifluoroacetic acid (TFA) and the deprotected compound **4** was coupled with the DOTA chelator. The crude product was purified using HPLC and the product eluate fraction was collected and lyophilized to obtain AV02053 in 10% yield (Table S1).

For the preparation of AV02070 (Scheme 2), methyl 2-chloroisonicotinate was coupled with *N*-Boc-*N*,*N*′-dimethyl-1,2-diaminoethane via nucleophilic substitution to obtain **5** in 8.4% yield. The methyl ester in **5** was hydrolyzed with NaOH in a mixture of water and methanol, leading to compound **6** in 90% yield. Compound **7** was obtained in 58% yield via esterification of compound **6** with TFP. The activated compound **7** was coupled with (*S*)-1-(2-aminoacetyl)-4,4-difluoropyrrolidine-2-carbonitrile to obtain compound **8** in 59% yield. Boc in compound **8** was removed using TFA, followed by coupling of the deprotected compound **8** with the DOTA chelator. The crude product was purified using HPLC and the product eluate fraction was collected and lyophilized to obtain AV02070 in 12% yield (Table S1).

Ga complexation of AV02053 and AV02070 was conducted in NaOAc buffer (0.1 M, pH 4.2–4.5) for nonradioactive standards and in HEPES buffer (2 M, pH 5.0) for ^68^Ga labeling, as previously reported (Tables S2 and S3) [32,33]. Ga-AV02053 and Ga-AV02070 were obtained in 42 and 22% yields, respectively. ^68^Ga-labeled AV02053 and AV02070 were obtained in 33–64% decay-corrected radiochemical yield with ≥44 GBq/µmol molar activity and >95% radiochemical purity.

### 2.2. Binding Affinity and Lipophilicity

**Figure 2:**
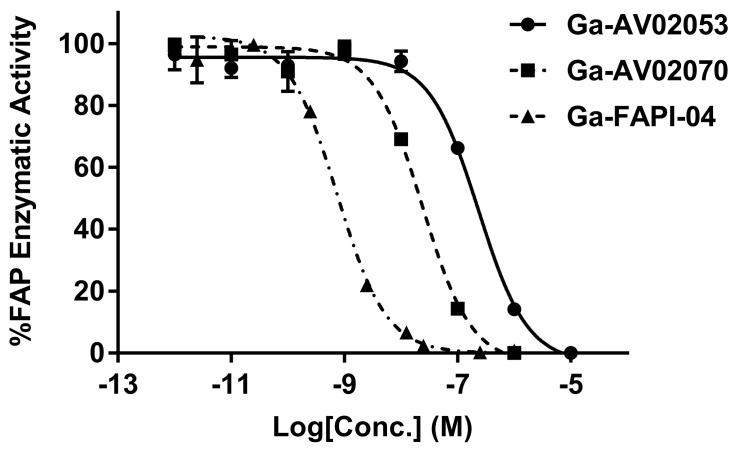
Inhibition of FAP enzymatic activity on Suc-Gly-Pro-AMC substrate by Ga-AV02053 and Ga-AV02070, and comparison with the data obtained previously using Ga-FAPI-04 [31]. The enzymatic activity represents the fluorescence of AMC which was cleaved by human FAP enzyme. The half-maximal inhibitory concentration (IC_50_) of the Ga-complex ligands was calculated, with Ga-FAPI-04 showing the lowest value, followed by Ga-AV02070 and Ga-AV02053. Error bars indicate standard deviations.

The binding affinities of Ga-AV02053 and Ga-AV02070 to human FAP were measured by an enzyme inhibition assay using Suc-Gly-Pro-AMC as the FAP substrate. The human FAP enzymatic activity on the substrate was inhibited by Ga-AV02053 and Ga-AV02070 in a dose dependent-manner (Figure 2). The calculated IC_50_ values for Ga-AV02053 and Ga-AV02070 were 187 ± 52.0 and 17.1 ± 4.60 nM, respectively. For comparison, the previously reported IC_50_ value for Ga-FAPI-04 under the same assay conditions was 1.03 ± 0.44 nM (Figure 2) [31].

The lipophilicity of ^68^Ga-labeled AV02053, AV02070, and FAPI-04 were estimated by measuring their LogD_7.4_ values using the shake flask method with n-octanol and phosphate-buffered saline (pH 7.4). The LogD_7.4_ values of [^68^Ga]Ga-AV02053, [^68^Ga]Ga-AV02070, and [^68^Ga]Ga-FAPI-04 were −3.75 ± 0.16, −3.45 ± 0.10, and −1.02 ± 0.35, respectively. The values indicate that these ^68^Ga-labeled tracers are hydrophilic, and the pyridine-based FAP-targeted tracers, [^68^Ga]Ga-AV02053 and [^68^Ga]Ga-AV02070, are more hydrophilic than [^68^Ga]Ga-FAPI-04.

### 2.3. PET Imaging, Ex Vivo Biodistribution, and Blocking Study

**Figure 3:**
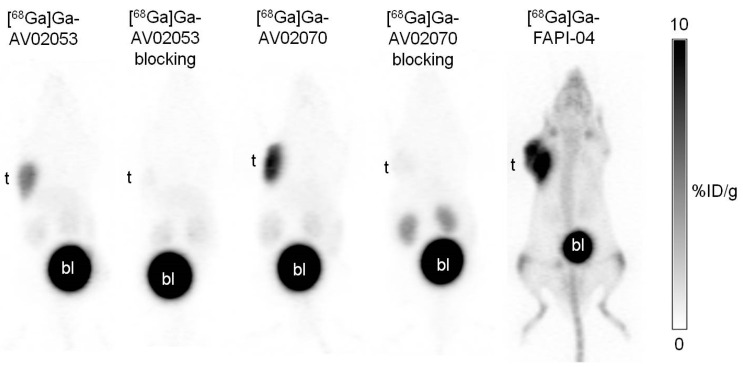
Representative maximum intensity projection PET images of [^68^Ga]Ga-AV02053 and [^68^Ga]Ga-AV02070 acquired at 1 h post-injection in mice bearing HEK293T:hFAP tumor xenografts. The image of [^68^Ga]Ga-FAPI-04 has been reported previously [31], and is displayed here for comparison. Despite having lower tumor uptake, [^68^Ga]Ga-AV02053 and [^68^Ga]Ga-AV02070 showed better tumor-to-background contrast compared to [^68^Ga]Ga-FAPI-04. The blocking with FAPI-04 decreased the tumor uptake of both [^68^Ga]Ga-AV02053 and [^68^Ga]Ga-AV02070 to background level. t: tumor; bl: urinary bladder.

Imaging studies showed that the HEK293T:hFAP tumor xenografts were clearly visualized in PET images acquired at 1 h post-injection using both [^68^Ga]Ga-AV02053 and [^68^Ga]Ga-AV02070 (Figure 3). Both tracers were excreted primarily through the renal pathway and had very low background uptake in normal organs/tissues. Although [^68^Ga]Ga-AV02053 and [^68^Ga]Ga-AV02070 had lower tumor uptake than that of previously reported [^68^Ga]Ga-FAPI-04, both of them had much lower background uptake, resulting in better tumor-to-background contrast than that of [^68^Ga]Ga-FAPI-04 (Figure 3). Co-injection of [^68^Ga]Ga-AV02053 and [^68^Ga]Ga-AV02070 with FAPI-04 (250 µg) reduced the uptake of [^68^Ga]Ga-AV02053 and [^68^Ga]Ga-AV02070 in HEK293T:hFAP tumor xenografts to almost background level, confirming the uptake of both tracers is FAP mediated.

**Figure 4:**
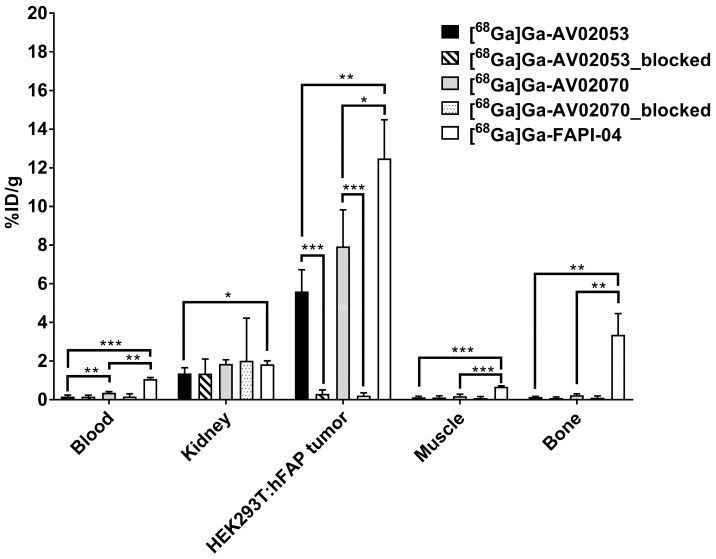
Biodistribution of [^68^Ga]Ga-AV02053 and [^68^Ga]Ga-AV02070 in HEK293T:hFAP tumor-bearing mice (n = 4) at 1 h post-injection with/without co-injection of FAPI-04 (250 µg). The biodistribution data for [^68^Ga]Ga-FAPI-04 have been reported previously [31]. Error bars indicate standard deviations. Statistical significance was calculated using unpaired two-tailed *t*-test with the Holm–Sidak method. * *p* < 0.05, ** *p* < 0.01, *** *p* < 0.001.

**Figure 5:**
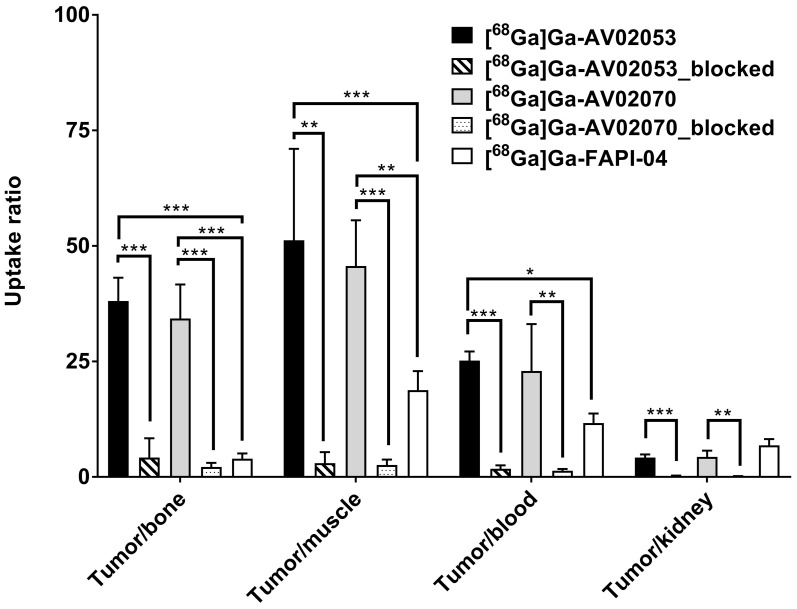
Tumor-to-background (bone, muscle, blood and kidney) uptake ratios of [^68^Ga]Ga-AV02053 and [^68^Ga]Ga-AV02070 in HEK293T:hFAP tumor-bearing mice (n = 4) at 1 h post-injection with/without co-injection of FAPI-04 (250 µg). The previously published data of [^68^Ga]Ga-FAPI-04 are presented for comparison [31]. Error bars indicate standard deviations. Statistical significance was calculated using unpaired two-tailed *t*-test with the Holm–Sidak method. * *p* < 0.05, ** *p* < 0.01, *** *p* < 0.001.

Biodistribution studies were conducted at 1 h post-injection with [^68^Ga]Ga-AV02053 and [^68^Ga]Ga-AV02070 in HEK293T:hFAP tumor-bearing mice, and the data are compared with those obtained previously using [^68^Ga]Ga-FAPI-04 (Figure 4 and Figure 5 and Table S4). The results are consistent with the observations from their PET images. Except tumor and kidneys (1.35–1.85%ID/g), the average uptake values of [^68^Ga]Ga-AV02053 and [^68^Ga]Ga-AV02070 in all other collected organs/tissues were <0.6%ID/g. The tumor uptake values of [^68^Ga]Ga-AV02053, [^68^Ga]Ga-AV02070, and [^68^Ga]Ga-FAPI-04 were 5.60 ± 1.12, 7.93 ± 1.88, and 12.5 ± 2.00%ID/g, respectively. Despite having a higher tumor uptake, [^68^Ga]Ga-FAPI-04 also had significantly higher blood, muscle, and bone uptake (1.07 ± 0.08, 0.67 ± 0.05 and 3.36 ± 1.09%ID/g, respectively) compared to those of [^68^Ga]Ga-AV02053 (0.22 ± 0.04, 0.12 ± 0.05 and 0.14 ± 0.02%ID/g, respectively) and [^68^Ga]Ga-AV02070 (0.36 ± 0.05, 0.19 ± 0.10 and 0.23 ± 0.06%ID/g, respectively). This also led to higher tumor-to-blood, tumor-to-muscle and tumor-to-bone uptake ratios for [^68^Ga]Ga-AV02053 (25.2 ± 1.97, 51.2 ± 19.8 and 38.1 ± 5.03, respectively) and [^68^Ga]Ga-AV02070 (22.9 ± 10.1, 45.7 ± 9.88 and 34.3 ± 7.35, respectively) than [^68^Ga]Ga-FAPI-04 (11.7 ± 2.04, 18.8 ± 4.09 and 3.93 ± 1.16, respectively) (*p* < 0.05).

Co-injection of FAPI-04 reduced the average uptake of [^68^Ga]Ga-AV02053 and [^68^Ga]Ga-AV02070 in HEK293T:hFAP tumor xenografts by 95% (5.60%ID/g down to 0.30%ID/g at 1 h post-injection) and 97% (7.93%ID/g down to 0.21%ID/g at 1 h post-injection), respectively, confirming the specific uptake of both tracers in tumors. On the contrary, there was no significant difference on the average uptake values of [^68^Ga]Ga-AV02053 and [^68^Ga]Ga-AV02070 in other major organs at 1 h post-injection with the co-injection of FAPI-04 (Figure 3 and Figure 4 and Table S4).

## 3. Discussion

Both AV02053 and AV02070 were prepared by multi-step organic synthesis approach, with overall unoptimized yields of 1.90% and 0.31%, respectively. We observed that the difference in the overall yield between AV02053 and AV02070 was mainly contributed by the efficiency of nucleophilic substitution of *N*-Boc-*N*,*N*′-dimethyl-1,2-diaminoethane with methyl 6-chloronicotinate (for the preparation of AV02053) and methyl 2-chloroisoncotinate (for the preparation of AV02070). The substitution of chloride in methyl 6-chloronicotinate (50% yield for compound **1**) is more efficient compared to methyl 2-chloroisoncotinate (8.4% yield for compound **5**). This is because the carbonyl group on the pyridine ring has better electron-withdrawing effect at the para position to chloride (in methyl 6-chloronicotinate) than at the meta position (in methyl 2-chloroisoncotinate).

The enzymatic assay (Figure 2) revealed that the FAP binding affinity of both Ga-AV02053 (IC_50_ = 187 ± 52.0 nM) and Ga-AV02070 (IC_50_ = 17.1 ± 4.60 nM) is lower than that of Ga-FAPI-04 (IC_50_ = 1.03 ± 0.44 nM). This is consistent with the observations by Jansen and co-workers that the quinoline-based pharmacophore (4-quinolinoyl)glycyl-(2*S*)-cyanopyrrolidine (IC_50_ = 10.3 nM) is more potent than its pyridine-based analog, (pyridine-4-carbonyl)glycyl-(2*S*)-cyanopyrrolidine (IC_50_ = 63 nM) [20]. We observed that the pyridine-4-carbonyl derived Ga-AV02070 (IC_50_ = 17.1 ± 4.60 nM) is more potent than its pyridine-3-carbonyl-derived analog, Ga-AV02053 (IC_50_ = 187 ± 52.0 nM). This is also consistent with the report by Poplawski and co-workers that (pyridine-4-carbonyl)-D-Ala-boroPro derivatives have better FAP binding affinity than their (pyridine-3-carbonyl)-D-Ala-boroPro analogs [21].

The LogD_7.4_ values confirmed that pyridine-based [^68^Ga]Ga-AV02053 and [^68^Ga]Ga-AV02070 are more hydrophilic compared to the quinoline-based [^68^Ga]Ga-FAPI-04 (LogD_7.4_ values = −3.75 ± 0.16, −3.45 ± 0.10 and −1.02 ± 0.35, respectively). The higher hydrophilicity and smaller size of the pyridine-based pharmacophores compared to the quinoline-based pharmacophores could potentially lead to FAP-targeted tracers with faster pharmacokinetics, resulting in lower background uptake and higher tumor-to-background image contrast.

Consistent with the predictions from their binding affinity and smaller molecular size of pharmacophores, PET imaging and biodistribution data (Figure 3, Figure 4 and Figure 5 and Table S4) revealed that the pyridine-based tracers, [^68^Ga]Ga-AV02070 (7.93 ± 1.88%ID/g) and [^68^Ga]Ga-AV02053 (5.60 ± 1.12%ID/g), have a lower tumor uptake than the previously reported value of the quinoline-based [^68^Ga]Ga-FAPI-04 (12.5 ± 2.00%ID/g) using the same HEK293T:hFAP tumor model [31]. However, the pyridine-based tracers have a lower uptake in muscle and bone, which are the two common off-target organs of FAP-targeted tracers, as observed in the quinoline-based [^68^Ga]Ga-FAPI-04 (Table S4). This leads to significantly higher tumor-to-muscle and tumor-to-bone uptake ratios for [^68^Ga]Ga-AV02070 and [^68^Ga]Ga-AV02053 than [^68^Ga]Ga-FAPI-04. The significantly lower blood uptake of [^68^Ga]Ga-AV02053 (0.22 ± 0.04%ID/g) and [^68^Ga]Ga-AV02070 (0.36 ± 0.05%ID/g) indicates that both tracers are rapidly cleared from the blood pool. Furthermore, the tumor uptake of both tracers was reduced by ≥95% with the co-injection of FAPI-04 (250 µg), demonstrating that tumor uptake of both tracers is FAP-mediated. The highly specific uptake of [^68^Ga]Ga-AV02070 and [^68^Ga]Ga-AV02053 in tumors and their superior tumor-to-background contrast suggest that both tracers are promising for clinical translation for cancer imaging.

There are some limitations in our research design: Firstly, the faster pharmacokinetics of pyridine-based FAP-targeted radioligands might limit their applications for radioligand therapy. This is because faster clearance from the blood pool reduces their chances to bind to FAP in tumors and might result in a relatively lower overall tumor uptake. This could be solved by the addition of an albumin binder to the pyridine-based FAP-targeted ligands to extend their blood residence time as similar approaches have been exploited to Increase the tumor uptake of radiolabeled quinoline-based FAPI-04 derivatives [34,35]. However, the blood residence time needs to be carefully adjusted as staying to long in blood will result in hematological toxicity. Previously, based on the reported albumin binder 4-(*p*-iodophenyl)butyramide [36], we have discovered a series of albumin binders with a broad range of albumin binding capability by replacing the iodo substituent with Br, Cl, F, H, CH_3_, NO_2_, OCH_3_, and NH_2_ [37]. These albumin binders could be used to fine-tune the blood residence time of pyridine-based FAP-targeted radioligands to maximize tumor uptake without inducing significant hematological toxicity.

Secondly, we hypothesized that the pyridine-based FAP-targeted tracers would have faster pharmacokinetics compared with the quinoline-based tracers. However, we did not conduct multiple time point imaging and biodistribution studies to investigate the pharmacokinetics of [^68^Ga]Ga-AV02053 and [^68^Ga]Ga-AV02070. Comparing the imaging and biodistribution data at 1 h post-injection, we clearly observed much lower background uptake of [^68^Ga]Ga-AV02053 and [^68^Ga]Ga-AV02070 when compared with those of ^68^Ga-FAPI-04, indicating that our pyridine-based tracers had faster pharmacokinetics. We did not specifically perform imaging and biodistribution studies at multiple time points mainly to minimize the number of used animals as this was considered as a pilot study. Once a promising candidate is identified, we will conduct multiple time point imaging and biodistribution studies to fully investigate its pharmacokinetics.

Thirdly, the phenomena observed in the preclinical mouse model might not be representative to what will be observed in the clinic. For example, the high bone uptake of [^68^Ga]Ga-FAPI-04 in mice is not observed in patients [23]. It will be of interest to compare [^68^Ga]Ga-FAPI-04 with [^68^Ga]Ga-AV02070 or [^68^Ga]Ga-AV02053 in the clinic to investigate if pyridine-based FAP-targeted tracers can still lead to better tumor-to-background contrast, and hence, a better detection sensitivity.

Lastly, the use of *N*,*N*′-dimethylethylenediamine as the linker between the DOTA chelator and the pyridine moiety of [^68^Ga]Ga-AV02070 and [^68^Ga]Ga-AV02053 (Figure 2), and the selected position for the linker to attach to the pyridine ring were only due to ease of synthesis and may not be optimal for FAP targeting. Further optimizations on the selection of linker as well as the position for the linker to attach to the pyridine ring might be needed to improve FAP binding affinity, tumor uptake, and maybe even the tumor-to-background contrast. A study reported by Lindner and co-workers [38] demonstrated that for the quinoline-based pharmacophores, a piperazine linker and attachment of the linker to the 6- rather than 7-position of the quinoline ring were preferable for FAP targeting. Both traits are preserved for the successful clinical tracers, [^68^Ga]Ga-FAPI-04 and [^68^Ga]Ga-FAPI-46 (Figure 2). Hence, for future modifications, we will investigate the effects of attaching the linker to the 2- vs. 3-position of the pyridine ring, as well as the use of a piperazine linker to potentially further increase binding affinity and tumor uptake.

## 4. Materials and Methods

### 4.1. Synthesis of Pyridine-Based FAP-Targeting Ligands

Detailed information for the synthesis, purification, and characterizations of AV02053 and AV02070, and their nonradioactive Ga-complexed standards and ^68^Ga-labeled analogs are provided in the Supplemental Information (Tables S1–S3).

### 4.2. Cell Culture

The HEK293T:hFAP cells generated in our lab [31] were cultured in DMEM GlutaMAX™ medium supplemented with 10% FBS, penicillin (100 U/mL) and streptomycin (100 μg/mL) at 37 °C in a Panasonic Healthcare (Tokyo, Japan) MCO-19AIC humidified incubator containing 5% CO_2_. Cells were grown until 80–90% confluence and washed with sterile phosphate-buffered saline (PBS, pH 7.4) and collected after 1 min trypsinization. The cell concentration was counted in triplicate using a hemocytometer and a manual laboratory counter.

### 4.3. In Vitro FAP Fluorescence Assay

The half maximal inhibitory concentration (IC_50_) values of the tested compounds for FAP were measured by in vitro enzymatic assay. The recombinant human FAP (Bio-legend, San Diego, CA, USA; 0.2 µg/mL, 50 µL) was added into costar 96-well plate. PBS and varied concentrations (0.2 pM to 2 µM) of tested nonradioactive Ga-complexed standards were added to each well (in duplicate) containing the recombinant human FAP. After being incubated for 30 min at 37 °C, 50 µL of Suc-Gly-Pro-AMC (2 µM, Bachem, Torrance, CA, USA) was added to each well. The fluorescent signals were acquired at 15, 30, 45, and 60 min using FlexStation 3 Multi-Mode Microplate Reader with excitation at 380 nm and emission at 460 nm. The IC_50_ (FAP) values were calculated using “nonlinear fit model” built-in model in GraphPad Prism 7.02 software.

### 4.4. LogD_7.4_ Measurement

The lipophilicity characteristics of the ^68^Ga-labeled pyridine-based FAPIs were determined by calculating the logarithm of the distribution coefficient (logD) in n-octanol/phosphate-buffered saline (PBS) of pH 7.4. Purified ^68^Ga-labeled tracer (50 µL) was added into a test tube containing 1 mL n-octanol and 1 mL PBS. The mixture was vortexed, followed by centrifugation for 5 min at 3000 rpm. The two layers were then collected separately, and the radioactivity was counted using a Perkin Elmer (Waltham, MA, USA) Wizard2 2480 automatic gamma counter. After adjusting the counts to background and calculating the ratio (D) of the activity of the organic to that of the aqueous phase, the logD values were then calculated.

### 4.5. Ex Vivo Biodistribution and PET/CT Imaging Studies

Imaging and biodistribution studies were performed using male NOD.Cg-Rag1tm1Mom Il2rgtm1Wjl/SzJ (NRG) mice following previously published procedures [39,40]. The experiments were conducted according to the guidelines established by the Canadian Council on Animal Care and approved by Animal Ethics Committee of the University of British Columbia. The mice were briefly sedated by inhalation of 2.5% isoflurane in oxygen, and HEK293T:hFAP cells (8.5 × 10^6^ cells) were inoculated subcutaneously behind the left shoulder. When the tumor grew to 5–8 mm in ~3 weeks, the mice were used for PET/CT imaging and biodistribution studies.

PET/CT imaging experiments were carried out using a Siemens (Knoxville, TN, USA) Inveon micro PET/CT scanner. Each tumor-bearing mouse was injected with ~4–6 MBq of ^68^Ga-labeled tracer through a lateral caudal tail vein under 2.5% isoflurane in oxygen anesthesia, followed by recovery and then roamed freely in its cage during the uptake period. At 50 min post-injection, a 10-min CT scan was conducted first for localization and attenuation correction after segmentation for reconstructing the PET images, followed by a 10-min static PET imaging acquisition.

For biodistribution studies, the mice were injected with the radiotracer (~2–4 MBq), as described above. For the blocking study, the mice were co-injected with 250 μg of FAPI-04. At 1 h post-injection, the mice were euthanized by CO_2_ inhalation. Blood was withdrawn by cardiac puncture, and organs/tissues of interest were collected, weighed, and counted using a Perkin Elmer (Waltham, MA, USA) Wizard2 2480 automatic gamma counter.

### 4.6. Statistical Analysis

Data were analyzed with the GraphPad Prism, version 7.02. Unpaired two-tailed t tests were performed for all organs in the biodistribution studies and blocking studies of [^68^Ga]Ga-AV02053, [^68^Ga]Ga-AV02070, and [^68^Ga]Ga-FAPI-04 in HEK293T:hFAP tumor models. A statistically significant difference was considered present when the adjusted *p* value was less than 0.05 using the Holm–Sidak method.

## 5. Conclusions

Two novel ^68^Ga-labeled pyridine-based FAP-targeted tracers were successfully synthesized and evaluated using a preclinical tumor model. Despite lower binding affinity and tumor uptake, both [^68^Ga]Ga-AV02070 and [^68^Ga]Ga-AV02053 show much higher tumor-to-background (blood, muscle, and bone) uptake ratios than [^68^Ga]Ga-FAPI-04. [^68^Ga]Ga-AV02070 containing a pyridine-4-carbonyl moiety has better binding affinity and tumor uptake than [^68^Ga]Ga-AV02053 containing a pyridine-3-carbonyl moiety, making it a promising candidate for the design of FAP-targeted tracers. Future optimization on the selection of linker between the DOTA chelator and the pyridine-based pharmacophores will be explored to increase the tumor uptake while maintaining or even further improving the high tumor-to-background contrast.

## Data Availability

The data generated from this study are available in the text and Supplementary Materials.

## Supplementary Materials

The following supporting information can be downloaded at: https://www.mdpi.com/article/10.3390/ph16030449/s1. Detailed synthetic procedures and results for the preparation of pyridine-based FAPI ligands, and their ^nat^Ga/^68^Ga-complexed analogs; Table S1: HPLC purification conditions and MS characterizations of DOTA-conjugated precursors; Table S2: HPLC purification conditions and MS characterizations of nonradioactive Ga-complexed standards; Table S3: HPLC conditions for the purification and quality control of ^68^Ga-labeled tracers; Table S4: Biodistribution and uptake ratios of ^68^Ga-labeled pyridine-based FAP-targeted tracers and FAPI-04 in HEK293T:hFAP tumor-bearing mice. Figure S1: Representative radio-chromatograms of [^68^Ga]Ga-AV02053 and [^68^Ga]Ga-AV02070 from QC HPLC.
