# Supplementary material for: Novel 68Ga-Labeled Pyridine-Based Fibroblast Activation Protein-Targeted Tracers with High Tumor-to-Background Contrast
Source: Pharmaceuticals (Basel). 2023 Mar 16;16(3):449. doi: 10.3390/ph16030449 (PMC10057391; doi:10.3390/ph16030449)
Supplement: Supplementary file 1 [file pharmaceuticals-16-00449-s001.zip › pharmaceuticals-2277554-supplementary.pdf]

SUPPLEMENTAL MATERIALS

General Methods

(*S*)-1-(2-aminoacetyl)-4,4-difluoropyrrolidine-2-carbonitrile 4-methylbenzenesulfonate was synthesized following literature procedure [1]. All other chemicals and solvents were obtained from commercial sources and used without further purification. Purification and quality control of DOTA-conjugated ligands and their $^{nat}Ga/^{68}Ga$-complexed analogs were performed on Agilent (Santa Clara, CA) HPLC systems equipped with a model 1200 quaternary pump, a model 1200 UV absorbance detector (220 nm), and a Bioscan (Washington, DC) NaI scintillation detector. The HPLC columns used were a semi-preparative column (Luna C18, 5 μm, 250 × 10 mm) and an analytical column (Luna C18, 5 μm, 250 × 4.6 mm) purchased from Phenomenex (Torrance, CA). The collected HPLC eluates containing the desired small molecules were lyophilized using a Labconco (Kansas City, MO) FreeZone 4.5 Plus freeze-drier. MS analyses were conducted using the Waters (Milford, MA) Acquity QDa mass spectrometer with the equipped 2489 UV/Vis detector and e2695 Separations module. C18 Sep-Pak cartridges (1 cm^3, 50 mg) were purchased from Waters (Milford, MA). ^{68}Ga was eluted from an ITM Medical Isotopes GmbH (Munich, Germany) generator, and purified according to the previously published procedures using a DGA resin column from Eichrom Technologies LLC (Lisle, IL) [2]. The radioactivity of ^{68}Ga-labeled tracers was measured using a Capintec (Ramsey, NJ) CRC®-25R/W dose calibrator and the radioactivity of mouse tissues collected from biodistribution studies were counted using a Perkin Elmer (Waltham, MA) Wizard2 2480 automatic gamma counter.

Synthesis of DOTA-conjugated Ligands

Synthesis of methyl 6-[[2-[[(*tert*-butoxy)carbonyl]methylamino]ethyl]methylamino]pyridine-3-carboxylate (**1**)

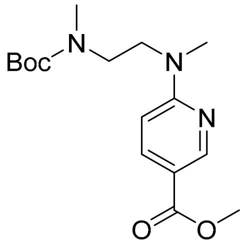

A solution of methyl 6-chloronicotinate (855 mg, 5.0 mmol), *N*-Boc-*N,N'*-dimethyl-1,2-diaminoethane (1.88 g, 10 mmol), and *N,N*-diisopropylethylamine (DIEA, 1.29 g, 10 mmol) in *N,N*-dimethylformamide (DMF, 50 mL) was stirred at 100 °C for 48 h. After evaporation, the residue was dissolved in diethyl ether (100 mL) and washed with water (100 mL). The organic phase was collected, dried over anhydrous $MgSO_4$, filtered, and evaporated under reduced pressure. The residue was purified with silica gel flash column chromatography eluted with 3:7 (v/v) ethyl acetate/hexanes. The product eluate fractions were combined, evaporated and dried under reduced pressure to yield **1** as a brown solid (803 mg, 50% yield). MS (ESI) calculated for $C_{16}H_{25}N_3O_4$ 323.2, found $[M+H]^+$ 324.2. ^1H NMR (300 MHz, $CDCl_3$) δ 8.78 (d, *J* = 2.3 Hz, 1H), 7.99 (d, *J* = 8.9 Hz, 1H), 6.46 (d, *J* = 9.0 Hz, 1H), 3.86 (s, 3H), 3.84 – 3.69 (m, 2H), 3.43 (t, *J* = 6.3 Hz, 2H), 3.11 (s, 3H), 2.86 (d, *J* = 17.7 Hz, 3H), 1.38 (s, 9H).

Synthesis of 6-[[2-[[(*tert*-butoxy)carbonyl]methylamino]ethyl]methylamino]pyridine-3-carboxylic acid (**2**)

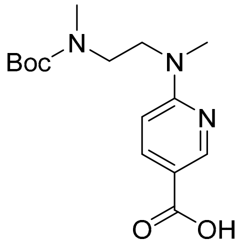

A solution of compound **1** (700 mg, 2.2 mmol) and sodium hydroxide (1.30 g, 32.3 mmol) in a mixture of water (15 mL) and methanol (20 mL) was stirred for 17 h. After evaporation, the residue was dissolved in water and the resulting solution was acidified with concentrated HCl to pH 3. The resulting white precipitate was filtered and dried under reduced pressure to yield **2** as a white powder (465 mg, 69% yield). MS (ESI) calculated for $C_{15}H_{23}N_3O_4$ 309.2, found $[M+H]^+$ 310.1. ^{1}H NMR (300 MHz, DMSO) δ 8.59 (d, *J* = 1.9 Hz, 1H), 7.89 (d, *J* = 8.8 Hz, 1H), 6.63 (t, *J* = 10.2 Hz, 1H), 3.72 (s, 2H), 3.04 (s, 2H), 2.75 (d, *J* = 13.2 Hz, 3H), 2.53 – 2.47 (m, 3H), 1.31 – 1.15 (m, 9H).

Synthesis of 2,3,5,6-tetrafluorophenyl 6-[[2-[[(*tert*-butoxy)carbonyl]methylamino]ethyl] methylamino]pyridine-3-carboxylate (**3**)

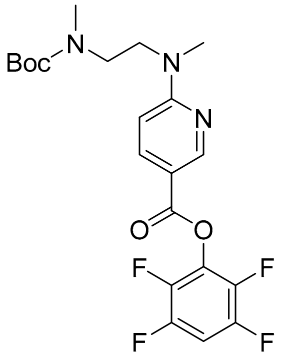

A solution of EDC·HCl (299 mg, 1.6 mmol) in DCM (12 mL) was added dropwise to a solution of compound **2** (440 mg, 1.4 mmol) and 2,3,5,6-tetrafluorophenol (307 mg, 1.9 mmol) in dichloromethane (DCM, 80 mL) cooled in an ice/water bath. The resulting mixture was stirred for 22 h. The solution was evaporated under reduced pressure and the residue was purified through silica gel flash column chromatography eluted with ethyl acetate. The product eluate fractions were combined, evaporated and dried under reduced pressure to yield **3** as a yellow oil (649 mg, 100% yield). MS (ESI) calculated for $C_{21}H_{23}F_4N_3O_4$ 457.2, found $[M+H]^+$ 458.2. ^{1}H NMR (300 MHz, $CDCl_3$) δ 8.92 (d, *J* = 5.2 Hz, 1H), 8.11 (t, *J* = 9.5 Hz, 1H), 7.01 (tt, *J* = 9.9, 7.0 Hz, 1H), 6.60 (s, 1H), 3.83 (dt, *J* = 15.4, 6.5 Hz, 2H), 3.50 (d, *J* = 6.6 Hz, 2H), 3.17 (s, 3H), 2.90 (d, *J* = 9.0 Hz, 3H), 1.40 (s, 9H).

Synthesis of (S)-N-(2-(2-cyano-4,4-difluoropyrrolidin-1-yl)-2-oxoethyl)-6-[[2-[[(*tert*-butoxy)-carbonyl]methylamino]ethyl]methylamino]pyridine-3-carboxamide (**4**)

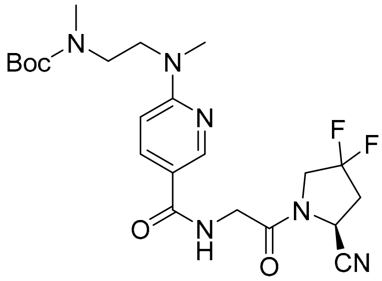

A solution of compound **3** (600 mg, 1.3 mmol), (*S*)-1-(2-aminoacetyl)-4,4-difluoropyrrolidine-2-carbonitrile 4-methylbenzenesulfonate (473 mg, 1.3 mmol) and triethylamine (TEA, 2.6 mmol, 265 mg) in acetonitrile (CH_3CN, 12 mL) was stirred at 50 °C for 22 h. The solution was then evaporated under reduced pressure and the residue was purified using silica gel flash column chromatography eluted with 1:9 (v/v) methanol/ethyl acetate. The product eluate fractions were collected, combined, evaporated and dried under reduced pressure to yield **4** as a light brown solid (365 mg, 55% yield). MS (ESI) calculated for $C_{22}H_{30}F_2N_6O_4$ 480.2, found $[M+H]^+$ 481.2. ^1H NMR (300 MHz, $CDCl_3$) δ 8.62 (d, *J* = 2.5 Hz, 1H), 7.86 (d, *J* = 8.7 Hz, 1H), 7,28(s, 1H), 6.43 (d, *J* = 9.0 Hz, 1H), 4.97 (t, *J* = 6.6 Hz, 1H), 4.38 (dd, *J* = 17.6, 5.5 Hz, 1H), 4.10 – 3.87 (m, 4H), 3.74 (m, 2H), 3.42 (t, *J* = 6.4 Hz, 2H), 3.11 (d, *J* = 8.6 Hz, 3H), 2.82 – 2.70 (m, 3H), 1.38 (s, 9H).

Synthesis of AV02053

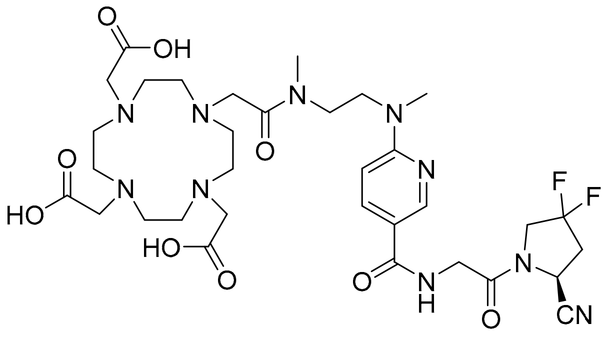

Trifluoroacetic acid (TFA, 1 mL) was added to a solution of compound **4** (21 mg, 44 µmol) in DCM (1 mL), and the resulting solution was stirred at room temperature for 2 h. The solution was then evaporated and the residue was dissolved in water (2 mL). DOTA-NHS-ester (40 mg, 53 µmol) was added, the pH of the resulting solution was adjusted to 8-9 with DIEA, and then the solution was stirred for 40 h. The crude solution was purified using HPLC and the eluted product fraction was collected and lyophilized to obtain AV02053 as a white powder (3.4 mg, 10% yield). MS (ESI) calculated for $C_{33}H_{48}F_2N_{10}O_9$ 766.4, found $[M+H]^+$ 767.2. The HPLC conditions and retention time for the purification of AV02053 are provided in Table S1.

Synthesis of methyl 2-[[2-[[(*tert*-butoxy)carbonyl]methylamino]ethyl]methylamino] pyridine-4-carboxylate (**5**)

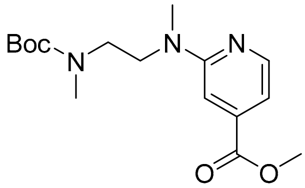

A solution of methyl 2-chloroisonicotinate (1.71 g, 10 mmol), *N*-Boc-*N,N'*-dimethyl-1,2-diaminoethane (3.25 g, 17 mmol), and DIEA (3.5 mL, 20 mmol) in DMF (60 mL) was stirred at 110 °C for 24 h. After evaporation, the residue was dissolved in diethyl ether (100 mL) and washed with water (100 mL). The organic phase was collected, dried over anhydrous $MgSO_4$, filtered, and evaporated under reduced pressure. The crude product was purified with silica gel flash column chromatography eluted with 1:3 (v/v) ethyl acetate/hexanes (1.6 L) followed by ethyl acetate (0.8 L). The product eluate fractions were combined, evaporated and dried under reduced pressure to yield **5** as a white solid (270 mg, 8.4% yield). MS (ESI) calculated for $C_{16}H_{25}N_3O_4$ 323.2, found $[M+H]^+$ 324.2. ^1H NMR (300 MHz, $CDCl_3$) δ 8.23 (d, *J* = 5.4 Hz, 1H), 7.04 (d, *J* = 5.4 Hz, 2H), 3.91 (s, 3H), 3.74 (dd, *J* = 15.0, 8.3 Hz, 2H), 3.42 (t, *J* = 6.3 Hz, 2H), 3.09 (s, 3H), 2.91 – 2.80 (m, 3H), 1.37 (d, *J* = 4.6 Hz, 9H).

Synthesis of 2-[[2-[[(*tert*-butoxy)carbonyl]methylamino]ethyl]methylamino]pyridine-4-carboxylic acid (**6**)

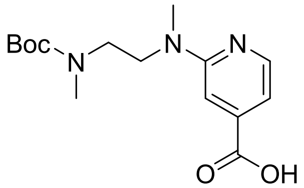

A solution of compound **5** (255 mg, 0.79 mmol) and sodium hydroxide (475 mg, 12 mmol) in a mixture of water (25 mL) and methanol (30 mL) was stirred for 20 h. After evaporation, the residue was dissolved in water and the resulting solution was acidified with concentrated HCl to pH 3. The resulting precipitates were collected by filtration and dried under reduced pressure to yield **6** as a light brown powder (220 mg, 90% yield). MS (ESI) calculated for $C_{15}H_{23}N_3O_4$ 309.2, found $[M+H]^+$ 310.2. ^1H NMR (300 MHz, $CDCl_3$) δ 8.20 (d, *J* = 5.01 Hz, 1H), 7.9 (d, *J* = 6.79 Hz, 2H), 3.39 (m, 2H), 3.04(s, 3H), 2.80 (d, J = 14.23 Hz, 2H), 2.11 (s, 3H), 1.31 (m, 9H).

Synthesis of 2,3,5,6-tetrafluorophenyl 2-[[2-[[(tert-butoxy)carbonyl]methylamino]ethyl] methylamino]pyridine-4-carboxylate (**7**)

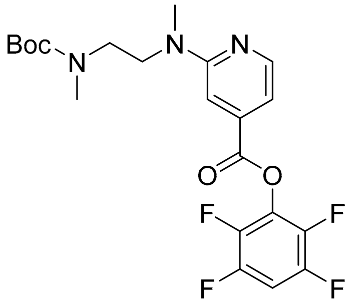

A solution of EDC·HCl (136 mg, 0.71 mmol) in DCM (10 mL) was added dropwise to a solution of compound **6** (200 mg, 0.65 mmol) and 2,3,5,6-tetrafluorophenol (139 mg, 0.84 mmol) in a mixture of DCM (30 mL) and DMF (10 mL) cooled in an ice/water bath. The resulting solution was stirred for 24 h and then evaporated. The residue was purified through silica gel flash column chromatography eluted with 1:2 (v/v) ethyl acetate/hexanes. The product eluate fractions were combined, evaporated and dried under reduced pressure to yield **7** as a yellow solid (170 mg, 58% yield). MS (ESI) calculated for $C_{21}H_{23}F_4N_3O_4$ 457.2, found $[M+H]^+$ 458.1.^{1}H NMR (300 MHz, $CDCl_3$) δ 8.32 (d, *J* = 5.2 Hz, 1H), 7.21 (d, *J* = 5.9 Hz, 2H), 7.06 (tt, *J* = 9.8, 7.0 Hz, 1H), 3.87 – 3.75 (m, 2H), 3.47 (t, *J* = 6.2 Hz, 2H), 3.14 (s, 3H), 2.91 – 2.89 (m, 3H), 1.37 (s, 9H).

Synthesis of (S)-N-(2-(2-cyano-4,4-difluoropyrrolidin-1-yl)-2-oxoethyl)-2-[[2-[[(*tert*-butoxy)-carbonyl]methylamino]ethyl]methylamino]pyridine-4-carboxamide (**8**)

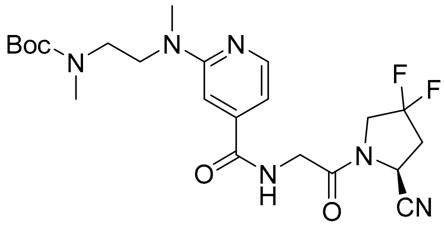

A solution of compound **7** (200 mg, 0.44 mmol), (*S*)-1-(2-aminoacetyl)-4,4-difluoropyrrolidine-2-carbonitrile 4-methylbenzenesulfonate (158 mg, 0.44 μmol) and TEA (88 mg, 0.88 mmol) in CH_3CN (5 mL) was stirred at 50 °C for 22 h. The solution was then evaporated under reduced pressure and the residue was purified using silica gel flash column chromatography eluted with 1:9 (v/v) methanol/ethyl acetate. The product eluate fractions were collected, combined, evaporated and dried under reduced pressure to yield **8** as a yellow solid (124 mg, 59% yield). MS (ESI) calculated for $C_{22}H_{30}F_2N_6O_4$ 480.2, found $[M+H]^+$ 481.2. ^{1}H NMR (300 MHz, $CDCl_3$) δ 8.12 (d, *J* = 5.0 Hz, 1H),), 7.23 (d, *J* = 20.8 Hz, 2H), 6.75 (d, *J* = 20.3 Hz, 1H), 4.91 (t, *J* = 6.6 Hz, 1H), 4.32 (d, *J* = 17.9 Hz, 1H), 3.97 (dd, *J* = 25.6, 13.1 Hz, 3H), 3.64 (t, *J* = 5.9 Hz, 2H), 3.42 – 3.28 (m, 2H), 2.99 (t, *J* = 19.4 Hz, 3H), 2.83 – 2.57 (m, 3H), 1.44 – 1.27 (m, 9H)

Synthesis of AV02070

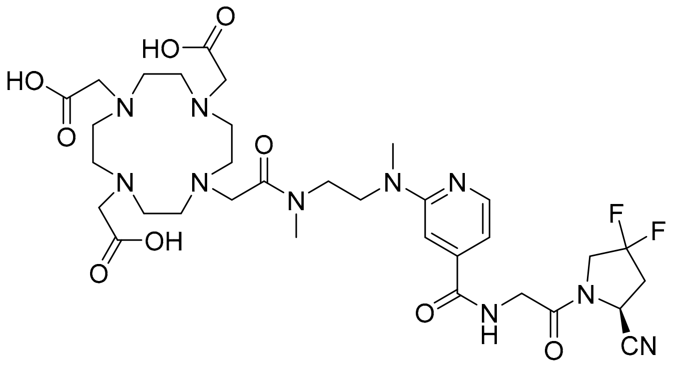

A solution of compound **8** (25 mg, 52 μmol) in DCM (1 mL) was added TFA and stirred at room temperature for 2 h. DOTA-NHS-ester (48 mg, 62 μmol) was added into the solution, the pH of the resulting solution was adjusted to 8-9 with DIEA, and then the solution was stirred for 40 h. The crude solution was purified using HPLC and the eluted product fraction was collected and lyophilized to obtain AV02070 as a white powder (4.6 mg, 12% yield). MS (ESI) calculated for $C_{33}H_{48}F_2N_{10}O_9$ 766.4, found $[M+H]^+$ 767.2. The HPLC conditions and retention time for the purification of AV02070 are provided in Table S1.

Synthesis of Nonradioactive Ga-complexed Standards

The nonradioactive Ga-complexed standards were prepared by reacting the DOTA-conjugated precursors with $GaCl_3$ (5 eq.) in NaOAc buffer (0.1 M, 500 μL, pH 4.2 – 4.5) at 80 °C for 15 min. The reaction mixture was then purified via HPLC (semi-preparative column, flow rate: 4.5 mL/min). The HPLC eluates containing the desired products were collected and lyophilized. The HPLC conditions, retention times, isolated yields and MS confirmations of these nonradioactive Ga-complexed standards are provided in Table S2.

Synthesis of ^{68}Ga-labeled Compounds

The radiolabeling experiments were performed according to previously published procedures [3-4]. Purified $^{68}GaCl_3$ in 0.5 mL water was added to a 4-mL glass vial preloaded with 0.7 mL of HEPES buffer (2 M, pH 5.0) and 10 μL precursor solution (1 mM). The radiolabeling reaction was carried out under microwave heating for 1 min, followed by purification using the semi-preparative HPLC column. The eluate fraction containing the radiolabeled product was collected, diluted with water (50 mL), and passed through a C18 Sep-Pak cartridge that was pre-washed with ethanol (10 mL) and water (10 mL). The C18 Sep-Pak cartridge was washed with water (10 mL), and the ^{68}Ga-labeled product was eluted off the cartridge with ethanol (0.4 mL). The eluted product was diluted with PBS for imaging and biodistribution studies. Quality control was performed using the analytical column. The HPLC conditions and retention times are provided in Table S3. The tracers were obtained in 33-64% decay-corrected radiochemical yields with ≥44 GBq/μmol molar activity and >95% radiochemical purity.

Table S1: HPLC purification conditions and MS characterizations of DOTA-conjugated precursors.

Compound name	HPLC conditions	Retention time (min)	Yield (%)	Calculated mass (m/z)	Found (m/z)
AV02053	15% CH_3CN and 0.1% TFA in H_2O	8.7	10	$[M+H]^+$ 767.4	$[M+H]^+$ 767.2
AV02070	14% CH_3CN and 0.1% TFA in H_2O	8.4	12	$[M+H]^+$ 767.4	$[M+H]^+$ 767.2

Table S2: HPLC purification conditions and MS characterizations of nonradioactive Ga-complexed standards.

Compound name	HPLC conditions	Retention time (min)	Yield (%)	Calculated mass (m/z)	Found (m/z)
Ga-AV02053	15% CH_3CN and 0.1% TFA in H_2O	7.2	42	$[M+2H]^{2+}$ 417.1	$[M+2H]^{2+}$ 417.3
Ga-AV02070	14% CH_3CN and 0.1% TFA in H_2O	6.8	22	$[M+2H]^{2+}$ 417.1	$[M+2H]^{2+}$ 417.4

Table S3: HPLC conditions for the purification and quality control of ^{68}Ga-labeled tracers. FA: formic acid.

Compound name	HPLC conditions		Retention time (min)
[^{68}Ga]Ga-AV02053	Semi-Prep	14% CH_3CN and 0.1% TFA in H_2O; flow rate 4.5 mL/min	13.4
	QC	15% CH_3CN and 0.1% TFA in H_2O; flow rate 2.0 mL/min	7.9
[^{68}Ga]Ga-AV02070	Semi-Prep	13% CH_3CN and 0.1% TFA in H_2O; flow rate 4.5 mL/min	15.5
	QC	16% CH_3CN and 0.1% TFA in H_2O; flow rate 2.0 mL/min	8.2

Table S4: Biodistribution (mean ± SD, n = 4) and uptake ratios of ^{68}Ga-labeled FAP-targeted tracers in HEK293T:hFAP tumor-bearing mice. The mice in the blocked group were co-injected with FAPI-04 (250 µg).

Tissue (%ID/g)	[^{68}Ga]Ga-AV02053		[^{68}Ga]Ga-AV02070		[^{68}Ga]Ga-FAPI-04*
	1 h	1 h blocked	1 h	1 h blocked	1 h
Blood	0.22 ± 0.04	0.15 ± 0.08	0.36 ± 0.05	0.16 ± 0.14	1.07 ± 0.08
Testes	0.10 ± 0.03	0.14 ± 0.11	0.17 ± 0.07	0.06 ± 0.05	0.28 ± 0.04
Small intestine	0.28 ± 0.07	0.27 ± 0.16	0.42 ± 0.13	0.35 ± 0.21	0.36 ± 0.08
Large intestine	0.08 ± 0.02	0.08 ± 0.06	0.30 ± 0.38	0.04 ± 0.03	-
Stomach	0.05 ± 0.02	0.03 ± 0.02	0.08 ± 0.04	0.06 ± 0.06	0.07 ± 0.01
Spleen	0.17 ± 0.06	0.14 ± 0.13	0.25 ± 0.12	0.07 ± 0.06	0.56 ± 0.11
Liver	0.52 ± 0.12	0.43 ± 0.24	0.39 ± 0.05	0.25 ± 0.14	0.36 ± 0.02
Pancreas	0.13 ± 0.04	0.10 ± 0.09	0.34 ± 0.45	0.05 ± 0.04	0.37 ± 0.05
Kidney	1.35 ± 0.29	1.35 ± 0.75	1.85 ± 0.21	2.01 ± 2.20	1.83 ± 0.16
Lungs	0.23 ± 0.04	0.20 ± 0.10	0.34 ± 0.06	0.17 ± 0.11	0.74 ± 0.11
Heart	0.07 ± 0.01	0.06 ± 0.03	0.12 ± 0.03	0.06 ± 0.05	0.34 ± 0.04
tumor	5.60 ± 1.12	0.30 ± 0.20	7.93 ± 1.88	0.21 ± 0.15	12.5 ± 2.00
Muscle	0.12 ± 0.05	0.11 ± 0.08	0.19 ± 0.10	0.08 ± 0.07	0.67 ± 0.05
Bone	0.14 ± 0.02	0.08 ± 0.06	0.23 ± 0.06	0.10 ± 0.09	3.36 ± 1.09
Brain	0.01 ± 0.00	0.01 ± 0.00	0.02 ± 0.02	0.01 ± 0.00	0.04 ± 0.00
Thyroid	0.12 ± 0.02	0.11 ± 0.12	0.22 ± 0.02	0.06 ± 0.04	-
Salivary glands	0.15 ± 0.07	0.12 ± 0.12	0.19 ± 0.06	0.11 ± 0.05	-
Tumor/bone	38.1 ± 5.03	4.18 ± 4.17	34.3 ± 7.35	2.15 ± 0.89	3.93 ± 1.16
Tumor/muscle	51.2 ± 19.8	2.97 ± 2.40	45.7 ± 9.88	2.59 ± 1.17	18.8 ± 4.09
Tumor/blood	25.2 ± 1.97	1.78 ± 0.73	22.9 ± 10.1	1.32 ± 0.41	11.7 ± 2.04
Tumor/kidney	4.19 ± 0.65	0.21 ± 0.09	4.34 ± 1.36	0.13 ± 0.04	6.85 ± 1.33

*The biodistribution data of [^{68}Ga]Ga-FAPI-04 have been reported previously [5].

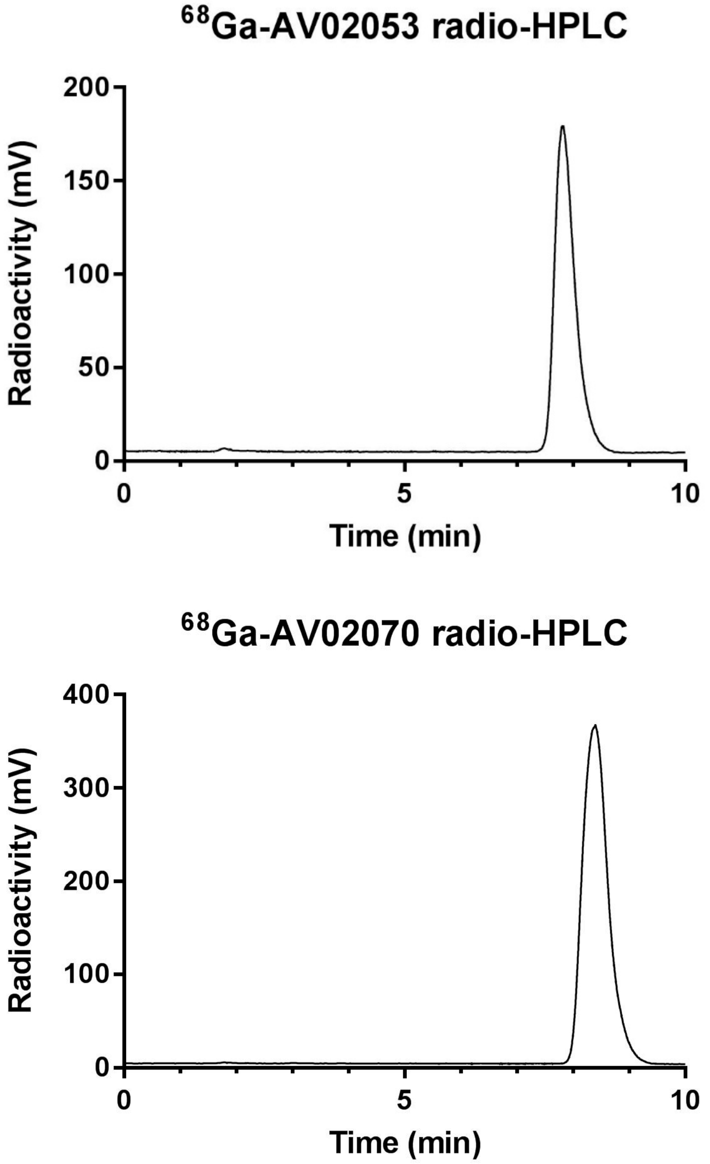

Figure S1. Representative radio-chromatograms of [^{68}Ga]Ga-AV02053 (top) and [^{68}Ga]Ga-AV02070 (bottom) from QC HPLC.

References

1. Jansen, K.; Heirbaut, L.; Verkerk, R.; Cheng, J. D.; Joossens, J.; Cos, P.; Maes, L.; Lambeir, A.-M.; De Meester, I.; Augustyns, K.; et al. Extended Structure–Activity Relationship and Pharmacokinetic Investigation of (4-Quinolinoyl)Glycyl-2-Cyanopyrrolidine Inhibitors of Fibroblast Activation Protein (FAP). *J. Med. Chem.* **2014**, *57*, 3053–3074.
2. Lin, K.-S.; Pan, J.; Amouroux, G.; Turashvili, G.; Mesak, F.; Hundal-Jabal, N.; Pourghiasian, M.; Lau, J.; Jenni, S.; Aparicio, S.; et al. In Vivo Radioimaging of Bradykinin Receptor B1, a Widely Overexpressed Molecule in Human Cancer. *Cancer Res.* **2015**, *75*, 387–393.
3. Zhang, C.; Zhang, Z.; Lin, K.-S.; Pan, J.; Dude, I.; Hundal-Jabal, N.; Colpo, N.; Bénard, F. Preclinical Melanoma Imaging with ^{68}Ga-Labeled α-Melanocyte-Stimulating Hormone Derivatives Using PET. *Theranostics* **2017**, *7*, 805–813.
4. Kuo, H.-T.; Lin, K.-S.; Zhang, Z.; Zhang, C.; Merkens, H.; Tan, R.; Roxin, A.; Uribe, C. F.; Bénard, F. What a Difference a Methylene Makes: Replacing Glu with Asp or Aad in the Lys-Urea-Glu Pharmacophore of PSMA-Targeting Radioligands to Reduce Kidney and Salivary Gland Uptake. *Theranostics* **2022**, *12*, 6179–6188.
5. Verena, A.; Zhang, Z.; Kuo, H.-T.; Merkens, H.; Zeisler, J.; Wilson, R.; Bendre, S.; Wong, A.A.W.L.; Bénard, F.; Lin, K.-S. Synthesis and Preclinical Evaluation of Three Novel ^{68}Ga-Labeled Bispecific PSMA/FAP-Targeting Tracers for Prostate Cancer Imaging. *Molecules* **2023**, *28*, 1088.
